# Effects of Thymol and Thymol α-D-Glucopyranoside on Intestinal Function and Microbiota of Weaned Pigs

**DOI:** 10.3390/ani10020329

**Published:** 2020-02-19

**Authors:** Noémie Van Noten, Jeroen Degroote, Elout Van Liefferinge, Bernard Taminiau, Stefaan De Smet, Tom Desmet, Joris Michiels

**Affiliations:** 1 Department of Animal Sciences and Aquatic Ecology, Ghent University, Coupure Links 653, 9000 Ghent, Belgium; noemie.vannoten@ugent.be (N.V.N.); jerdgroo.degroote@ugent.be (J.D.); elout.vanliefferinge@ugent.be (E.V.L.); stefaan.desmet@ugent.be (S.D.S.); 2 Department of Food Sciences, University of Liège, Quartier Vallée 2, Avenue de Cureghem 7A-7D, 4000 Liège, Belgium; bernard.taminiau@uliege.be; 3 Department of Biotechnology, Ghent University, Coupure Links 653, 9000 Ghent, Belgium; tom.desmet@ugent.be

**Keywords:** thymol α-D-glucopyranoside, thymol, weaned piglets, microbiota

## Abstract

**Simple Summary:**

Weaning is a stressful event for piglets reared under commercial conditions, often resulting in economic losses due to reduced animal performance and health. The growing discouragement of antibiotic applications under these circumstances has stimulated the search for alternatives, like plant-derived products, to sustain piglet health. Thymol, the main compound in thyme essential oil, is considered as a valid alternative, mainly due to its antimicrobial properties. However, upon ingestion thymol quickly disappears from the upper gastro-intestinal tract, so that the concentrations remaining in the distal small intestine are far too low to kill off undesired bacteria. We tested gluco-conjugation, the linkage of a compound with a glucose unit, as a protective measure to obtain elevated thymol concentrations in the gut. Therefore, weaner piglets were fed a basal diet either un-supplemented or supplemented with pure thymol or its gluco-conjugate, thymol α-D-glucopyranoside. Neither treatment could change the microbial composition. Nevertheless, thymol reduced diarrhea incidence and improved intestinal integrity, while thymol α-D-glucopyranoside did not. To conclude, gluco-conjugation insufficiently protected thymol from fast absorption and negated the positive physiological effects of thymol, indicating that further research is warranted.

**Abstract:**

The present study evaluated gluco-conjugation as a measure to delay thymol absorption and enhance its antimicrobial activity in the gut of weaned piglets. The three dietary treatments consisted of a basal diet without additives (T_CON_), supplemented with thymol at 3.7 mmol/kg dry matter (T_THY_), or with an equimolar amount of thymol α-D-glucopyranoside (T_TαG_). Each dietary treatment was replicated in 6 pens with 2 piglets per pen (*n* = 12 for analytical parameters) and was supplemented for 14 days. The total (free plus gluco-conjugated) thymol concentrations in the stomach contents were 14% lower in T_TαG_ as compared to T_THY_ piglets. Neither of the additives could be detected further down the gut. *E.coli* counts in the proximal small intestine were significantly lower in T_THY_ than in T_TαG_ pigs (3.35 vs. 4.29 log_10_ CFU/g); however, other bacterial counts and their metabolites were unaffected by treatment. A metagenomic bacterial analysis revealed a great relative abundance of *Lactobacillus* spp. in the distal small intestine (range 88.4–99.9%), irrespective of treatment. The intestinal barrier function was improved by T_THY_, but not T_TαG_, compared to T_CON._ In conclusion, gluco-conjugation did not result in higher thymol concentrations in the gut, but conversely, it seemed to diminish the biological effects of thymol in vivo.

## 1. Introduction

The common practice of early weaning (usually around 3–5 weeks of age) in commercial pig production subjects the immature piglets to a variety of stressors caused by alterations in the social structure, diet, and environment [1]. Therefore, weaning negatively impacts piglet health, performance, and sometimes survival rates, which eventually results in heavy economic losses [2]. Because many countries are now restricting the use of antibiotics and pharmacological zinc oxide in animal production [3,4,5], there is a growing need for cost-effective alternatives to control post-weaning diarrhea and improve the performance of weaned piglets.

Phytogenic feed additives are widely recognized as promising candidates to replace in-feed antibiotics [6]. They are mainly engaged to stabilize the gastro-intestinal (GI) functions with the ultimate goal to improve animal performance [7]. One of the most frequently investigated functional phytochemicals is thymol, the main monoterpene phenol in the essential oil (EO) extracted from thyme, which possesses antimicrobial, anti-oxidative, and anti-inflammatory properties [8]. These features make thymol a promising bioactive substance for application in animal feed, but the implementation still encounters some challenges [9]. Although EOs are mainly applied for their antimicrobial effects [6], the recommended doses of commercially available products are far below the required minimal inhibitory concentrations (MIC) for the modulation of gut microbiota [10,11]. Moreover, the lipophilic nature of EOs, including thymol, impedes the effective delivery of the active compounds to the gut, due to their fast and almost complete absorption in the stomach and proximal small intestine [12]. Increasing inclusion levels in the diet is not recommended with regard to cost-effectiveness [9], reduced palatability [13], and possible toxic effects [14]. Therefore, it is vital to find proper protection methods in order to obtain adequate antimicrobial concentrations in the distal small intestine, where most diarrhea-causing enterotoxigenic *Escherichia coli* reside [15].

Glyco-conjugation is a process that naturally occurs in plants to increase the water solubility and stability of secondary metabolites and to alter their functionality [16]. Moreover, the potential of glycoside prodrugs has been explored in human medicine as well [17]. However, when orally administering these glyco-conjugates, hydrolysis in the GI tract should take place to release the active aglycon [17]. Based on ex vivo tests with everted porcine jejunal segments, Petrujkić et al. (2013) [18] concluded that thymol β-D-glucopyranoside (TβG) was more resistant to intestinal absorption than its aglycon, thymol. Although we were unable to demonstrate the delayed absorption of TβG or its stereo-isomer thymol α-D-glucopyranoside (TαG) after supplementing piglets for one day in our previous experiment [19], it still remains to be elucidated what the long-term effects of TαG supplementation are. Indeed, the absorbed thymol might display beneficial effects via the systemic route or it might exert topical action on the epithelial mucosa, where it has been shown to accumulate [20,21]. Furthermore, gluco-conjugation has the additional advantage of reducing the volatility [22] and masking the pungent taste of thymol [23]. Therefore, this research investigated whether the long term supplementation of weaned piglets with TαG could increase the luminal thymol concentrations in the small intestine. Furthermore, we aimed to elaborate on the effects of this TαG supplementation on the performance, small intestinal barrier function, and gut microbiota composition, as compared to un-supplemented or thymol-fed animals.

## 2. Materials and Methods

### 2.1. Animals and Housing

The study was conducted in accordance with the ethical standards and recommendations for accommodation and care of laboratory animals covered by the European Directive (2010/63/EU) on the protection of animals used for scientific purposes and by the Belgian royal decree (KB29.05.13) on the use of animals for experimental studies. The experiment did not involve interventions causing harm equivalent to, or higher than, that caused by the introduction of a needle in accordance with good veterinary practice, and because animals were killed solely for the use of their organs or tissues (2010/63/EU). Moreover, electronarcosis followed by exsanguination is an approved method for euthanasia for this animal species. For this experiment, 36 piglets (Topigs hybrid × Piétrain) were selected from 12 litters of a commercial farm. At weaning (28 days of age), three healthy, median weight piglets (range 6.5 ± 1.5 kg) were selected from each litter. Both castrated males and females were included. The selected piglets were transported to the trial facilities, where they were housed per two per pen (2.10 m^2^/pen) on full slatted floors. Until d5 post-weaning, the ambient temperature in the stable was kept at 30 °C with 24 h light. From d6 till d15, the ambient temperature was linearly adjusted to 28 °C with a 18L:6D light schedule. The animals had *ad libitum* access to feed and water at all times. The basal diet, from which the three experimental diets were derived, was wheat and barley based and formulated to meet or exceed recommendations by the Dutch Centraal Veevoederbureau [24]. More details about the composition of the diet can be found in Table 1. The nutrient composition was analyzed according to the methods described by Spranghers et al. (2018) [25].

### 2.2. Experimental Design and Measurements

The experiment comprised three dietary treatments: the basal diet without additives (T_CON_), supplemented with thymol (T_THY_) at 3.7 mmol/kg dry matter (DM; equivalent to 3.3 mmol/kg or 500 mg/kg as fed), or with an equimolar amount of thymol α-D-glucopyranoside (T_TαG_). The experimental diets were prepared by dividing one batch of basal diet in three equal parts and mixing the appropriate amounts of the additives into the feed. Thymol was a crystalline powder and could be mixed into the feed as such, while TαG was mashed with a mortar to obtain a fine granular product prior to mixing. No carriers or other protection methods, like coating, were used. Subsamples of each diet were gathered immediately after mixing and stored at −20 °C for the analytical verification of the additives. Thymol (CAS: 89-83-8; purity: 99.5%) was obtained from Sigma Aldrich (Bornem, Belgium). TαG (79% purity, 0.6% free thymol, 3.5% thymol diglucoside) was enzymatically synthesized according to the procedure described in De Winter et al. (2015) [26] from a reaction mixture containing thymol (5 g/L), sucrose (1 mol/L), and facilitated by the R134A mutant of *Thermoanaerobacterium thermosaccharolyticum* sucrose phosphorylase (4 U/mL). 

Treatments were replicated in six pens with two piglets per pen. First, piglets were allocated to the pens in order to stratify for mean body weight and litter origin. Therefore, three littermate piglets were assigned to three neighboring pens, and this was repeated with three piglets from a second litter. This process was repeated six times. Next, the filled 18 pens were assigned to the three treatments according to a randomized block design, where each treatment was randomly assigned to two pens per block. In this way, every treatment included six males and six females originating from 12 sows (Figure S1). The piglets were weighed individually on d0, d5, and d13 post-weaning. The feeders were weighed daily to determine the feed intake at the pen level. The average daily gain (ADG), average daily feed intake (ADFI), and feed:gain ratio (F:G) were calculated for the periods d0–5, d5–13, and d0–13 post-weaning. The same trained animal caretaker inspected the animals daily for general health, fecal consistency, and diarrhea incidence. The fecal consistency score was visually assessed on the pen level according to the following scoring system: 1 = hard or slightly moist feces, clearly formed, normal; 2 = moist or soft feces, but still with a definite form, sticky; and 3 = watery or liquid feces, unformed, diarrhea. The assessment of diarrhea incidence was done simultaneously by counting the piglets in a pen receiving a fecal score of 3 that showed clear signs of diarrhea, i.e., filthy, wet backside and tail, dehydrated, loss of condition, and irritation of the skin around the anus. The incidence was calculated at the pen level as the number of times a fecal score of 3 was given over the whole trial divided by the number of ‘animal days’ (i.e., number of animals in a pen (2) multiplied by the number of days that they were present (13)). 

### 2.3. Sample Collection

The sample collection was spread over three days (d13-15 post-weaning) for practical reasons. Per sampling day, 12 piglets (four per treatment) were brought to electronarcosis followed by exsanguination. The entire GI tract was removed, exposed, and partitioned in the following six compartments for digesta collection: stomach, three parts of the small intestine (SI1, SI2, and SI3 corresponding to segments of 0–25%, 25–75%, and 75–100% of the total length, respectively), caecum, and mid-colon. The contents of each compartment were quantitatively collected and stirred well. A fresh aliquot (approximately one gram) of SI1 and SI3 was processed for bacteriological plating. Next, two digesta subsamples were taken from every compartment. The first was acidified to pH < 2 with 2% of 6 M H_2_SO_4_ to stop fermentation and stored at −20 °C pending an analysis of active compounds and bacterial metabolites. The second subsample was snap frozen in liquid nitrogen and stored at −80 °C for the determination of the glycosidase activity. A last aliquot of SI3 digesta was collected in a sterile tube, snap frozen, and stored at −80 °C to determine the bacterial composition. The remaining digesta were frozen and freeze-dried for the determination of the dry matter contents. Meanwhile, a first 5 cm section of the small intestine was obtained at 75% (distal jejunum) of the total length, rinsed with 0.9% saline, and fixated in a 4% formaldehyde solution for a histo-morphological analysis. A second segment (20 cm) was obtained at 75% to assess the macromolecular permeability in the Ussing chambers (only the first two sampling days for practical reasons). Finally, a third segment (20 cm) was collected at 2.5% (duodenum), 25% (proximal jejunum), and 75% for its mucosa, harvested by gently scraping the mucosal surface with a glass slide, and was stored at −80 °C pending an analysis of the glycosidase activity. 

### 2.4. Laboratory Analysis

#### 2.4.1. Concentrations of Active Compounds in Digesta

The concentration of the active compounds, thymol, and TαG in digesta samples of different GI compartments was determined as described by Van Noten et al. (2020) [19]. The samples were extracted in ethyl acetate, 1-butanol, and 1-propanol (60:30:10; *v*/*v*/*v*) containing 2-isopropylphenol (0.25 g/L) and 4-nitrophenyl α-D-glucopyranoside (0.05 g/L) as internal standards for thymol and TαG, respectively. The organic phase was evaporated to dryness under nitrogen gas, and the residue was reconstituted in a mixture of water and acetonitrile (65:35; *v*/*v*). The extracted samples were subjected to an HPLC analysis with an elution gradient on a reversed phase C18 column and with the detection of the active compounds at 280 nm.

#### 2.4.2. Small Intestinal Barrier Function and Histo-Morphology

The intestinal permeability was assessed using the Ussing chamber technique, as previously described by Wang et al. (2016) [27]. In brief, the segment of the distal jejunum was rinsed with saline, stripped from its outer muscle layers, slit longitudinally, and mounted into inserts with an exposed tissue area of 1.07 cm². Two replicate chambers were used per pig, and the tissues were mounted within 10 min *post-mortem*. Fluorescein isothiocyanate–dextran 4-kDa (FD4; Sigma-Aldrich, Bornem, Belgium) and horseradish peroxidase 40-kDa (HRP; type IV; Sigma-Aldrich) were used as macromolecular probes. The markers were added to the mucosal side after 20 min of equilibration to obtain final concentrations of 0.8 mg/mL and 0.4 mg/mL, respectively. Samples were taken from the serosal side every 20 min between 40 min and 100 min after mounting the segments. The fluorescent signal of FD4 was captured using an excitation filter at λ = 494 nm and an emission filter at λ = 521 nm. An analysis of the HRP activity was performed, as described by McKie et al. (1999) [28], but the measurement was performed in a kinetic way (10 cycles, 1-min interval).

The histo-morphological parameters villus height (VH) and crypt depth (CD) were determined according to standard procedures including dehydration, embedding in paraffin, and mounting of 5 µm transverse sections on glass slides [29]. After staining with hematoxylin-eosin, the VH and CD were measured in at least 10 well-oriented villi and adjacent crypts using an Olympus BX61 microscope and image analysis software (analySIS Pro, Olympus, Aartselaar, Belgium). VH:CD was calculated as the mean value of the ratios of the obtained villus heights and adjacent crypt depths.

#### 2.4.3. Bacteriological Analyses

The bacterial counts (viable counts; log_10_ CFU /g fresh digesta) in the digesta of SI1 and SI3 were obtained using the ring-plate technique [30]. Seven serial 10-fold dilutions were made from 1 g of fresh digesta in a sterilized peptone solution (peptone, 1 g/L; agar, 0.4 g/L; NaCl, 8.5 g/L; and cysteine, 0.7 g/L) and plated onto selective media for counting the following bacterial groups: lactobacilli (Rogosa Agar, CM0627B, Oxoid, Basingstoke, UK; incubated for 48 h at 37 °C under anaerobic conditions), total anaerobic bacteria (Reinforced Clostridial Agar, CM0151B, Oxoid; incubated for 48 h at 37 °C anaerobic conditions), *Escherichia coli* (Tryptone Bile X-Glucuronide Agar, CM0945B, Oxoid; incubated aerobically for 24 h at 37 °C), coliform bacteria (Eosin Methylene Blue Agar, CM0069B, Oxoid; incubated aerobically for 24 h at 37 °C), and streptococci (Slanetz & Bartley Medium, CM0377B, Oxoid; incubated aerobically for 48 h at 37 °C). 

Bacterial metabolites were determined in the digesta of SI3 and the mid-colon. The volatile fatty acids (VFA) and lactic acid in the small intestinal contents were analyzed simultaneously as described by Missotten et al. (2009) [31]. Briefly, 2-ethyl butyric acid was added to the acidified samples as an internal standard. The mixture was extracted with diethyl ether, followed by derivatization with N-tert-butyldimethylsilyl-N-methyltrifluroacetamide and a subsequent analysis on GC. A VFA analysis of mid-colon samples was performed on GC after extraction with 10% formic acid with ethyl butyric acid as the internal standard, as described by Castro-Montoya et al. (2012) [32]. The lactic acid content in the digesta from the mid-colon was quantified according to the microdiffusion method of Conway (1957) [33]. In short, lactic acid is oxidized by cerium to acetaldehyde, which then reacts with semi-carbazide to form a semi-carbazon. The latter is measured with a spectrophotometer at 224 nm.

The microbial composition analysis was performed by 16S ribosomal RNA (rRNA) profiling in the SI3 digesta. First, genomic DNA was extracted using PSP Spin Stool DNA Plus Kit (Invitek, Westburg, Netherlands) according to the manufacturer’s instructions. Next, the 16S polymerase chain reaction (PCR) libraries were generated and processed as described by Cong et al. (2019) [34]. In short, the V1-V3 hypervariable region of the bacterial 16S rRNA was amplified using (5′-GAGAGTTTGATYMTGGCTCAG-3′) and (5′-ACCGCGGCTGCTGGCAC-3′) as forward and reverse primers, respectively. After purification, amplicons were sequenced on the Illumina Miseq platform (Illumina, San Diego, USA) with the V3 chemistry kit. The obtained sequences were processed using MOTHUR (software package v1.39.5) for alignment and clustering [35], while the VSEARCH algorithm [36] was utilized for chimera detection. The final reads were clustered in operational taxonomic units (OTU) with a distance unit cut-off of 0.03. The SILVA database (v1.28) of full-length 16S rRNA sequences [37] served as the basis for reference alignment and taxonomical assignment using the BLASTN algorithm. Originally, 6,567,253 raw reads were obtained. After cleaning for length and sequence quality, 6,204,369 reads remained, and the removal of chimeric contaminants resulted in 6,059,497 reads (49,734–287,387 reads per sample). Subsampled datasets of 10,000 reads per sample were utilized for further analysis. The alpha diversity was evaluated with MOTHUR at the genus level by calculating the Chao1 richness index (richness), reciprocal Simpson biodiversity index (diversity), and Simpson evenness index (evenness). All raw reads of small intestinal digesta samples have been deposited at the National Center for Biotechnology Information (NCBI) and are available under the BioProject ID: PRJNA603645.

#### 2.4.4. Glycosidase Activity in Mucosa and Digesta

Prior to the quantification of the glycosidase activity in mucosa, phosphate buffered extracts were made. Therefore, one gram of mucosa was homogenized with 5 mL ice-cold phosphate buffered saline (PBS) (0.1 mol/L, pH 7.0) using an Ultra Turrax T25 (IKA) at 9500 rpm for 30 s. The mixture was centrifuged (15,000× *g*, 15 min, 4 °C), and the supernatant was divided into small portions and stored at −80 °C until analysis. During the extraction process, samples and supernatants were kept on ice all the time to prevent enzyme degradation. The glycosidase activity was determined spectrophotometrically by measuring the appearance of p-nitrophenol (pNP) after the hydrolysis of its respective p-nitrophenyl glycoside. Three activities were evaluated: α-glucosidase (αGLU), β-glucosidase (βGLU), and β-galactosidase (βGAL). All substrates (p-nitrophenyl α-D-glucopyranoside (EN03227), p-nitrophenyl β-D-glucopyranoside (EN03181) and p-nitrophenyl β-D-galactopyranoside (EN06672), respectively) were purchased from Carbosynth Ltd., Compton Berkshire, UK. Substrates were dissolved in PBS, and the resulting stock solutions (13.3 mmol/L) were pre-incubated at 37 °C. The final reaction mixtures (pH 7.0) were prepared in 96-well plates by mixing 1 volume of supernatant with 3 volumes of the appropriate substrate. The well-plate was immediately transferred to the absorbance reader (Tecan Infinite M Nano) where the release of pNP was determined at 405 nm and at 37 °C during nine kinetic cycles with an interval of 90 s. All samples were analyzed in triplicate. 

Digesta homogenates were prepared in a similar way (except: 1 g sample in 4 mL PBS), but an extra filtration step of the supernatants over a paper filter (particle retention: 12–15 µm) was performed. The stock solutions contained 20 mmol pNP-substrate/L, and the reaction mixture was obtained by mixing 1 volume of substrate solution with 1 volume of supernatant. A standard curve of pNP dissolved in the same PBS buffer (pH 7.0) was used to calculate the glycosidase activity, which was expressed in units, with one unit (U) corresponding with 1 mmol of pNP released per min per g protein or with 1 nmol of pNP released per min per g fresh matter, for mucosa and digesta, respectively. 

The bicinchoninic acid method [38] was used to determine the protein content of the mucosal extracts (sample to standard working reagent-ratio = 1:8; incubation: 30 min at 37 °C). The standard was bovine serum albumin. All chemicals were bought from Sigma Aldrich (Bornem, Belgium).

#### 2.4.5. Statistical Analysis

The experimental unit for animal performances was the pen. The individual animal was considered the experimental unit for analytical variables. All statistical analyses, except for the 16S microbial composition data, were performed with SPSS statistics 25.0 program (SPSS Inc., Chicago, IL, USA). Assumptions of normality and equality of variances were checked using the Shapiro-Wilkinson and Levene’s test, respectively, before an analysis of the data by the general linear model (GLM) procedure with treatment as a fixed factor and block as a random factor, in combination with Tukey post-hoc tests. The blocking factor accounted for variability between litters and sampling days. In case of heteroscedasticity, the one-way ANOVA procedure with Welch correction was used in combination with Games-Howell post-hoc tests. In case the data were not normally distributed, Kruskal-Wallis non-parametric tests in combination with Dunn’s post-hoc tests were used. Concentrations of active compounds in digesta were subjected to t-tests to discern differences with feed concentrations. Differences were considered significant at *p* ≤ 0.05, and statistical tendencies were assumed when 0.05 < *p* < 0.10. The data are presented as least square means with SEM, unless stated otherwise.

Statistical differences in microbial richness, diversity, and population evenness between the treatments on the genus level were investigated using the GLM procedure, with treatment as a fixed factor and block as a random factor. The microbial composition data were analyzed on the genus level with MOTHUR to assess the diversity clustering of dietary treatments with the Bray-Curtis matrix using the analysis of molecular variance (AMOVA) and the homogeneity of molecular variance (HOMOVA). The AMOVA test is a non-parametric analysis for testing the hypothesis that the genetic diversity within one treatment is not significantly different from the genetic diversity in all treatments together. The HOMOVA test is a non-parametric analysis used to test the hypothesis that the genetic diversity within the different treatments is homogeneous [39]. Statistical differences in relative bacterial abundances between treatments were tested by non-parametric Kruskal–Wallis tests with Tukey-Kramer post-hoc tests and the Benjamin–Hochberg false discovery rate using the STAMP software (Version 2.1.3) on the phylum, family, and genus levels.

## 3. Results

### 3.1. Animal Performance

No animals had to be removed from the study, no mortality occurred during the experiment, and antibiotic treatments were limited to a few individual intramuscular injections. No significant differences were found in body weight (BW), ADG, ADFI, or F:G between the dietary treatments in any of the monitored periods post-weaning (Table 2). Piglets from the control treatment lost weight during the first 5 days post-weaning, whereas the supplemented groups displayed a slightly positive ADG in this period (*p* > 0.05). The ADFI did not differ significantly between treatments in any of the monitored periods post-weaning. However, piglets from treatment T_TαG_ performed numerically the best as to BW, ADG, and ADFI during the whole experimental period (*p* > 0.05). On the other hand, there was a tendency (*p* = 0.082) for a lower F:G in TαG (−4.67) as compared to thymol (5.06) fed pigs from d0–5 post-weaning. However, this was due to two T_TαG_-pens which had a normal ADFI (62 and 82 g), but a slightly negative ADG (−3 and −5 g, respectively). The diarrhea incidence was highest between d4 and d7 post-weaning (data not shown). The average fecal consistency score was significantly lower in thymol-fed piglets (1.53) as compared to control animals (1.81) on a scale from 1 (firm) to 3 (watery), with an intermediate score for TαG-fed piglets (1.60). Moreover, there was also a tendency (*p* = 0.086) for a lower diarrhea incidence in T_THY_ (12.2%) as compared to T_CON_ (24.3%).

### 3.2. Concentrations of Active Compounds

**Figure 1:**
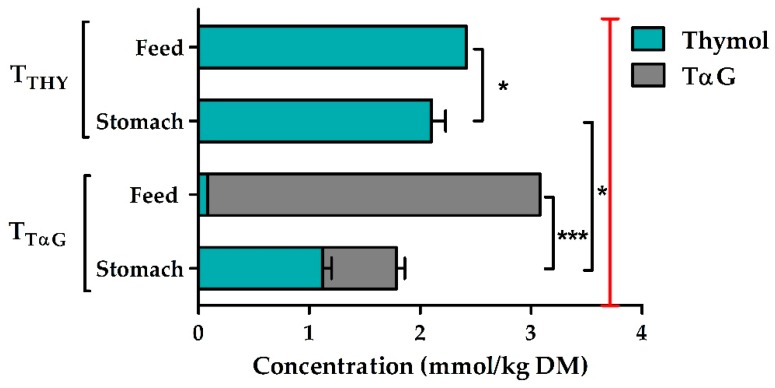
The concentration of thymol and thymol α-D-glucopyranoside (TαG) in the feeds and stomach digesta of piglets fed an intended dose of 3.7 mmol/kg DM (vertical red line) of thymol (T_THY_) or TαG (T_TαG_).

The analytically verified total thymol concentration measured in the feeds differed from the intended dose of 3.7 mmol/kg DM. T_THY_ feeds contained 2.4 mmol thymol/kg DM, while T_TαG_ feeds included 3.0 mmol TαG /kg DM and 0.08 mmol thymol/kg DM (Figure 1). Further calculations were made with these actual concentrations. The thymol concentration in the stomach contents of T_THY_ pigs was 13% lower than in the feed (on DM basis) (*p* = 0.031).

Furthermore, in the stomach contents of T_TαG_ pigs only 37% of the thymol was present as gluco-conjugate, while the remaining 63% was present in its free form. In addition, in the stomach the remaining total thymol concentration (sum of free and conjugated form) was only 58% of the feed concentration for the T_TαG_ treatment (*p* < 0.001). Finally, the total thymol concentration was significantly lower in the stomach contents of T_TαG_ piglets (1.78 mmol/kg DM) as compared to T_THY_ animals (2.10 mmol/kg DM), even though the actual concentration in the feed was lower in the T_THY_ treatment. The concentrations of both active compounds in the small intestine, caecum, and colon were below the detection limit and thus not quantifiable.

### 3.3. Small Intestinal Barrier Function and Histo-Morphology

Supplementing thymol in the diet of piglets significantly reduced the jejunal permeability for FD4 with 56% as compared to the control treatment (Table 3). The permeability of HRP, on the other hand, was unaffected by treatment. Regarding the histo-morphological parameters, there was a tendency (*p* = 0.071) for an increased VH:CD ratio in T_THY_ when compared to T_CON_. This difference was not due to changes in the crypt depth, but could be explained by the numerically longer villi (*p* > 0.05).

### 3.4. Bacteriological Analyses 

#### 3.4.1. Bacterial Counts

The counts of different bacterial groups in the digesta of the proximal (SI1) and distal small intestine (SI3) are listed in Table 4. 

At the level of the proximal small intestine, the supplementation of thymol significantly reduced the numbers of *E. coli* with almost one log unit as compared with TαG (3.35 versus 4.29 log_10_ CFU/g, respectively). Other bacterial counts in the proximal small intestine remained unchanged. Similarly, no major changes in bacterial counts were induced by dietary supplementation in the distal small intestine. However, the numbers of Lactobacilli (*p* = 0.070), *E. coli (p* = 0.078) and total coliforms (*p* = 0.069) tended to be lower in T_THY_ than in T_TαG_, while there was a trend (*p* = 0.069) towards lower counts of total anaerobes in thymol-supplemented groups as compared to control piglets.

#### 3.4.2. Bacterial Metabolites

Concentrations of bacterial metabolites in the contents of the distal small intestine and mid-colon did not differ amongst treatment groups (Table 5). However, there was more of a tendency (*p* = 0.088) towards lower valerate percentages in the mid-colon of thymol-supplemented groups than in control groups.

#### 3.4.3. Microbial Composition by 16S rRNA Profiling

**Figure 2:**
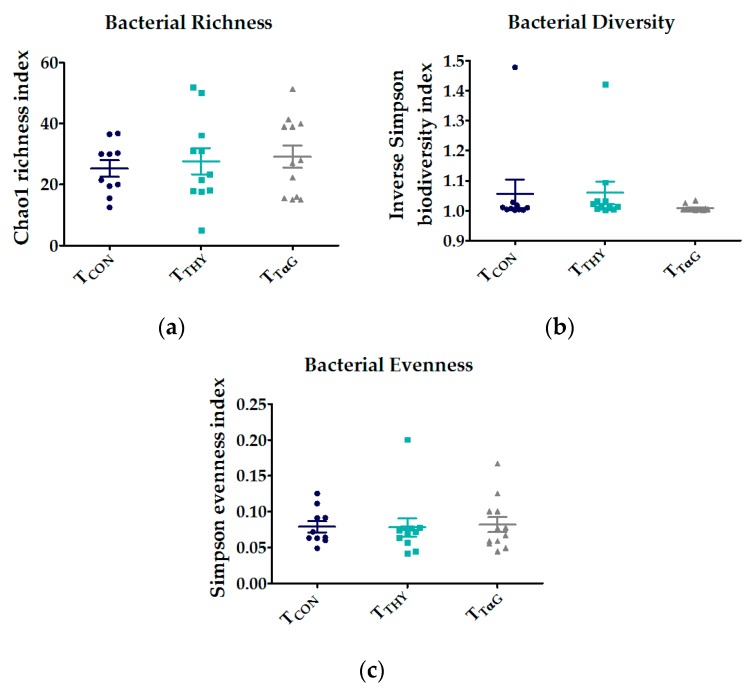
The alpha diversity indices of the microbial community composition on the genus level in the small intestinal contents of the last 25% of the total length of piglets fed a basal diet (T_CON_) supplemented with 3.7 mmol/kg DM thymol (T_THY_) or thymol α-D-glucopyranoside (T_TαG_) (**a**) bacterial richness (Chao1 richness index); (**b**) bacterial diversity (Reciprocal Simpson biodiversity index; and (**c**) bacterial evenness (Simpson evenness index). The data are represented as scatter plots: each dot represents a piglet, together with the mean (middle line) and SEM (whiskers).

Due to insufficient intestinal material, only 10 samples (out of 12) of the T_CON_ group could be sequenced. Additionally, one piglet from the T_THY_ group was removed from the dataset because of a deviating microbial profile (see piglet 22 in Figure S2). Thus, in total 33 16S metagenomic profiles were used (10 T_CON_, 11 T_THY_, and 12 T_TαG_), in which 678 different OTUs were identified. No treatment effects were present on the alpha diversity of the communities in SI3 (Figure 2) as estimated by Chao1 (*p* = 0.621), reciprocal Simpson (*p* = 0.551), and Simpson evenness indexes (*p* = 0.906). Additionally, the beta diversity analysis using AMOVA and HOMOVA did not reveal significant differences in the community composition between treatments (*p* = 0.166 and *p* = 0.341, respectively). 

**Figure 3:**
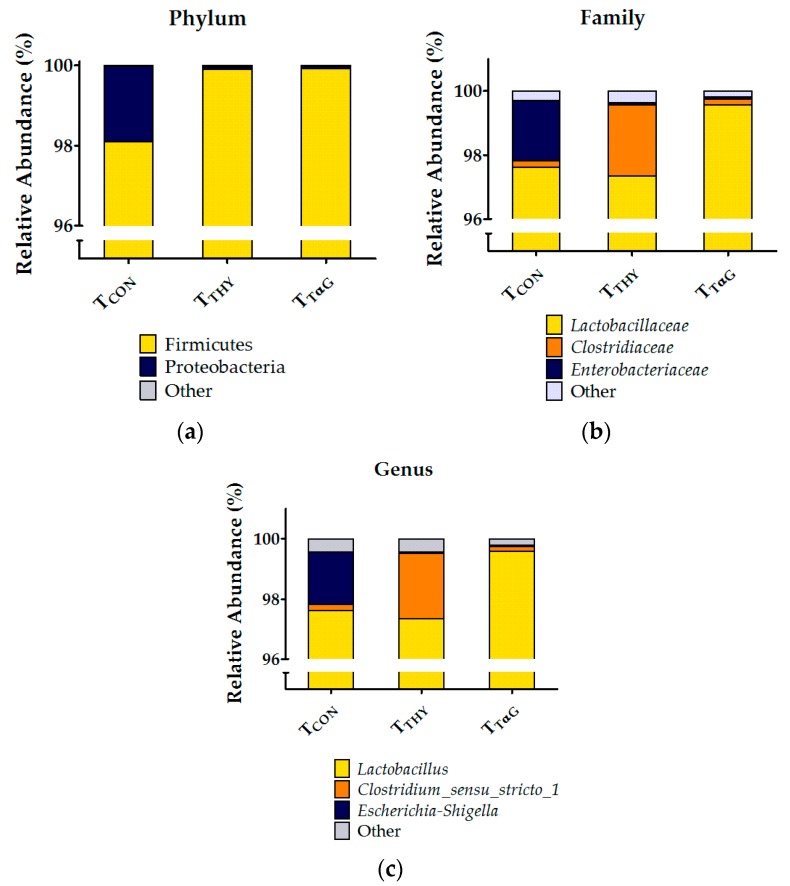
The cumulated relative abundances of the identified taxa at the (**a**) phylum, (**b**) family, and (**c**) genus levels in the small intestinal contents of the last 25% of the total length of piglets fed a basal diet (T_CON_) supplemented with 3.7 mmol/kg DM thymol (T_THY_) or thymol α-D-glucopyranoside (T_TαG_).

The cumulated relative abundances of the microbial community in SI3 at the phylum, family, and genus levels are represented in Figure 3, while the microbial community compositions at the same levels of each individual piglet are available in Figure S2. Across all diet groups, 10 phyla were identified, with Firmicutes being an extremely dominant phylum (98.1–99.9% of the total sequences). The second largest phylum was Proteobacteria, accounting for 0.06–1.89% of the sequences, and all the other phyla had a relative abundance below 0.01%. At the family level, it became evident that these Firmicutes are mainly represented by *Lactobacillaceae* and *Clostridiaceae*, accounting for 97.3–99.6% and 0.2–2.2% of the total sequences, respectively, while *Enterobacteriaceae* are the main family representing the Proteobacteria, with 0.06–1.88% of the sequences. The other 52 identified families had relative abundances below 0.1%. The major genera were *Lactobacillus* (97.3–99.6%), *Clostridium*_sensu_stricto_1 (0.2–2.2%), and *Escherichia-Shigella* (0.05–1.76%). Furthermore, the relative abundances of the remaining 119 identified genera were below 0.1%. Finally, *Lactobacillus amylovorus* was identified as the most abundant species, accounting for 91.9–95.4% of all identified sequences. Other abundant species were an uncultured *Lactobacillus_*Otu00010 (2.4–3.0%), *Clostridium*_sensu_stricto_1_DQ797692 (0.12–2.05%), *Escherichia coli* (0.03–1.68%), and *Lactobacillus delbrueckii* (0.24–0.52%). No statistical differences were found among the treatments on the relative abundance of taxonomic groups at the phylum, family, or genus levels. Because the dominance of lactobacilli might have masked more subtle treatment-induced variations in other taxa, an attempt was made to remove those sequences identified as *Lactobacillus* on the genus level from the dataset before subsampling. Unfortunately, fewer than 10,000 sequences remained for the majority of the samples, and a large variation in the sequencing depth was created between samples, making a statistical analysis insufficiently reliable. 

### 3.5. Glycosidase Activity in Mucosa and Digesta

The βGLU and βGAL activities in the duodenal mucosa were significantly reduced by 34% and 30%, respectively, in thymol-supplemented piglets as compared to control animals (Table 6). This effect, however, did not persist more distally in the small intestine, nor was the αGLU activity affected by treatment. In the digesta, no treatment effects could be demonstrated for any of the measured glycosidase activities in either compartment.

## 4. Discussion

### 4.1. Effect of Thymol and TαG Supplementation on Pig Performance

Although numerous trials have evaluated the effects of herbs and EO mixtures containing thymol on pig performance with variable outcomes [40,41,42], to our knowledge no data are available concerning the consequences of TαG supplementation. Additionally, only a limited amount of pig experiments reporting performance data have been carried out with pure thymol, and in none of these cases supplementation (range 67–10,000 mg thymol/kg feed) resulted in significantly increased ADG or final BWs [43,44,45,46]. This is in agreement with our findings, although thymol- and TαG-supplemented piglets performed numerically better than the control in the current trial.

The lack of statistical significance is probably due to the low number of replicates (*n* = 6) for performance parameters. This was, however, not the focus of our study. Besides, Trevisi et al. (2007) [43] reported a reduced ADFI in piglets supplemented with 10,000 mg thymol/kg due to the negative impact of this high dose on the palatability. Still, our results showed no differences in feed intake between treatments, indicating that piglets had no aversion towards pure or glycosylated thymol. Similarly, Anderson et al. (2012) [46] and Michiels et al. (2010) [44] described no alterations in the feed intake at low (67–201 mg/kg) and fairly high (500–2000 mg/kg) thymol doses, respectively.

Even though no significant improvements in growth performance were demonstrated in the current study, thymol supplementation did result in firmer feces and tended (*p* = 0.086) to reduce the diarrhea incidence in comparison to control piglets. This could indicate a beneficial modulation of the intestinal microbiota, although microbial metabolites were unaffected in the small and large intestines. Unfortunately, no compositional analysis was performed for the colon. Otherwise, improved fecal scores potentially relate to the improved barrier function in the small intestine, which might indicate a reduced inflammatory status of thymol-supplemented pigs [47].

### 4.2. Effects of TαG Supplementation on Thymol Concentrations in the Proximal GI Tract

The suitability of EOs or their compounds as feed additives also depends on their stability during feed processing and storage [48]. In the current study, there was quite a large difference between the intended dose (3.7 mmol/kg DM) and the analytically verified concentrations in the feed, with recoveries of 65% and 81% for thymol and TαG, respectively. This discrepancy might have been due to the volatility of thymol; hence, evaporation might have taken place while mixing it into the feed [9]. This would also explain the higher recovery of TαG, as gluco-conjugation reduces the volatility of phenolics. Next, thymol and other EO compounds might absorb hydrophobic components in the feed, which not only reduces their antimicrobial activity [49] but also makes it harder to recover them from the complex feed matrix. However, the recovery rates of thymol and TαG from the feed matrix of the applied extraction method were verified for different concentrations and ranged between 101% and 122% [19]. Although the final feed concentrations were considerably lower than expected, the concentrations were still manifold higher than the concentrations of commercial products currently applied in animal feeds [50].

It was demonstrated by Michiels et al. (2008) [12] that, upon ingestion, thymol is quickly absorbed in the stomach and the proximal small intestine. Indeed, in-feed supplementation of thymol at 500 or 2000 mg/kg for 12 days yielded roughly four times lower concentrations (expressed as mg/kg fresh digesta) at the level of the stomach [44]. This is in agreement with our current results, taking into account the analytically verified thymol concentration in the feed (325 mg/kg feed) and a mean thymol concentration in the stomach contents of 96 ± 6.2 mg/kg fresh digesta in thymol-treated piglets. However, we preferred to express our results on a molar and dry matter basis; the latter to rule out the dilution effect of consumed water and the mixing with digestive juices. Then, the thymol concentration was only 13% lower in the stomach than in the feed. Still, from the duodenum on, thymol levels were no longer detectable, indicating a very quick disappearance from the intestinal lumen. Next, gluco-conjugation was examined as a protective measure against fast absorption, as was suggested by Petrujkić et al. (2013) [18]. In their ex vivo experiments conducted with TβG in everted porcine jejunal segments, the gluco-conjugate was absorbed 2.3 to 2.8 times less efficiently as compared to free thymol [18]. Unfortunately, our data could not confirm these findings. On the contrary, TαG supplementation led to 15% lower total thymol concentrations in the stomach contents than the addition of pure thymol. Additionally, more than half of the TαG in the stomach was already hydrolyzed into free thymol, and no detectable levels of either active compounds could be demonstrated in the small and large intestinal contents of T_TαG_ animals. These results are in agreement with our previous experiment, where piglets were only supplemented for one day [19]. Still, it remains to be elucidated whether thymol glucosides can be absorbed through the gastric wall or if prior hydrolysis is required. Some flavonoid glycosides, such as those of quercetin [51] and daidzein [52], are not absorbed intact from the stomach, while anthocynanins (glyco-conjugated anthocyanidins) can cross the gastric wall [53]. In any case, linking thymol with one glucose unit did not result in higher luminal thymol concentrations in the distal small intestine, suggesting that conjugation with more complex sugars or longer sugar chains would be opportune. Indeed, Cermak et al. (2003) [54] demonstrated that metabolites of rutin, the glucorhamnoside of quercetin, appeared 2 h later in the blood of pigs than those of the monoglucoside after oral administration, indicating delayed absorption. Nevertheless, in our case, the absorbed thymol might still have an advantageous effect via the systemic route. Moreover, the monoterpene might exert a topical action on the epithelial mucosa, as thymol has been shown to accumulate on the mucosal tissues [20,21].

### 4.3. Effects of Thymol and TαG Supplementation on the Intestinal Barrier Function

Under healthy conditions, the intestinal epithelium functions as a selective barrier that inhibits the permeation of pathogens and antigens into the mucosal tissues, while allowing the passage of beneficial molecules like nutrients, ions, and water [55]. Tight junction (TJ) proteins are crucial to maintaining the barrier function, as they link adjacent epithelial cells on the apical side, thus controlling the paracellular transport route in a dynamic way [7,55]. Essential oils can regulate the barrier function by acting upon the gene expression and synthesis of TJ proteins [7]. Indeed, it was demonstrated in vitro that pretreatment of IPEC-J2 cells with thymol (50 µM) could alleviate the negative effects of lipopolysaccharide-induced damage to the barrier function by increasing the trans-epithelial electrical resistance and reducing the FD4 flux [47]. Likewise, pigs fed a diet with oregano EO (25 mg/kg) during 4 weeks showed higher jejunal expression levels of the TJ proteins occludin and zonula occludens-1 and had longer villi, implying an improved intestinal integrity [56]. In agreement with these findings, our data also suggest an enhanced barrier function in the distal jejunum of thymol- but not TαG-supplemented piglets. This was apparent from the decreased FD4 flux and the tendency for an increased VH:CD ratio in the T_THY_ treatment. The transcellular absorption route, however, was not affected, as can be seen from the unaltered HRP flux. It remains unclear why the gluco-conjugated form of thymol was unable to strengthen the GI integrity, especially because the luminal concentrations of both thymol and TαG in the distal jejunum were too low (<10 mg/kg) to be reliably detected with the current analytical method. Still, the effective thymol concentration needed to enhance the barrier function might be below our detection limit, as Omonijo et al. (2019) [47] found already increased TEER values at only 50 µM or 7.5 mg/kg. 

### 4.4. Effects of Thymol and TαG Supplementation on Gut Microbiota

The application of thymol in pig feeds, in pure form or as a blend, has shown varying degrees of success with regard to the modulation of gut microbiota ([42,44,45,46,56,57]. In our study, the supplementation of thymol or TαG had no effect on the bacterial counts or their metabolites in the distal small intestine. Similarly, the bacterial metabolites in the mid-colon were unaltered. The lack of microbiota-modulating effects in the distal GI tract is probably due to the fact that the trace amounts of the active compounds (<10 mg/kg) remaining in those compartments were far below the required MICs for commensals and pathogens [10,11]. However, in the proximal small intestine, the *E. coli* counts were almost one log_10_ CFU/g lower in thymol- than in TαG-treated pigs, although the active compounds were no longer quantifiable in this compartment as well. It is supposed that the thymol supply from the stomach must have been sufficiently high to exert its antimicrobial action against *E.coli* before being absorbed, whether or not in combination with a potential topical action of thymol adhered to the mucosa. Furthermore, the lack of antimicrobial effects in the proximal small intestine following TαG supplementation might be caused by several factors. First, the supply of free and/or conjugated thymol from the stomach into the duodenum was potentially too low, especially when compared with the levels of active compounds retained in the stomach of thymol-fed piglets. Next, upon arrival of TαG in the duodenum, the presence of the sodium-dependent glucose transporter 1 (SGLT1) might facilitate the fast absorption of the intact gluco-conjugate [58]. Eventually, the glucosidase capacity in the duodenum might have been insufficient to hydrolyze TαG at an adequate rate into thymol plus glucose. Indeed, gluco-conjugation impairs the antimicrobial action of thymol, and hydrolysis by intestinal bacteria [59,60] or brush border enzymes like lactase phlorezin hydrolase [58] is necessary to release the bactericidal aglycon.

Furthermore, a bacterial population analysis of the distal small intestine using 16S rRNA profiling was unable to highlight any diet-induced differences in the microbiome in the current study. Likewise, Li et al. (2018) [61] did not find differences in bacterial diversity or richness indices in the colon after supplementation of a carvacrol-thymol blend. However, these authors did find a shift in the relative abundance at the phylum level from Bacteroidetes to Firmicutes in the colonic contents of EO-supplemented piglets [61]. The contrast with our results is probably due to the difference in gut location and the extremely high relative abundance of Firmicutes (>98%) in the control group, not leaving much space for an additional increase in relative numbers. Indeed, clear differences exist between the microbial communities of the small and large intestine. The jejunum and ileum show a lower bacterial diversity and an enrichment of Firmicutes relative to the large intestinal contents [62] and feces [63]. Proteobacteria was the second most abundant phylum in our samples, after Firmicutes. This observation is largely in agreement with the literature, although the reported relative abundances of these phyla fluctuate with values roughly ranging between 60% and 95% for Firmicutes and between 2% and 30% for Proteobacteria [62,63,64,65]. A variety of factors might explain the discrepancy between studies, such as differences in diets, antimicrobial use, age of the animals, breed, and environment [34]. Looft et al. (2014) [62] ascribed the difference between ileal and colonic microbiota to the dominance of *Anaerobacter* and *Turicibacter* in the ileum. However, these genera were hardly detected in our samples, while the most abundant genus was undoubtedly *Lactobacillus* (>97%). This is in agreement with the results of Argüello et al. (2018) [63] who found *Lactobacillus* levels of about 60% in 5 week-old piglets. Lactobacilli might have been stimulated by the lactose present in the diet (4%), as these bacteria are able to use the simple milk sugar for their energy production [66]. Finally, in agreement with our findings, *L. amylovorus*-like phenotypes were reported to be an abundant member of the ileal lactobacillus community [67,68]. Their presence is considered beneficial because of its antimicrobial activity against enteric pathogens [69]. In conclusion, notwithstanding that the desired modulating effect on the microbiota was absent, the supplementation of weaned piglets with thymol or TαG did not cause dysbiosis in the distal small intestine.

### 4.5. Effects of Thymol and TαG Supplementation on the Glycosidase Activity in Mucosa

Several studies have demonstrated the potential of EOs to enhance nutrient digestibility by promoting digestive secretions [6]. Digestibility was mainly increased for dry matter, proteins, and amino-acids, rather than for starch [70,71,72]. Although EO supplementation did increase the activities of pancreatic α-amylase and intestinal maltase in broiler chickens [73], we demonstrated an inhibitory effect of thymol supplementation on the βGLU and βGAL activity in the duodenal mucosa of piglets, which might interfere with the digestion of dietary sugars, like lactose, or other glucosides present in the diet, e.g., genistein or daidzin from soybeans [74]. Gluco-conjugation weakened the suppressive effect of thymol. In vitro studies have demonstrated the competitive inhibition of αGLU and βGAL by phytogenic compounds like isoquercitrin [75], carvacrol [76], and thymol [77]. However, further research is warranted to elucidate the mechanisms by which thymol inhibits glycosidase activity in vivo, as the literature data on this specific aspect are very limited. 

## 5. Conclusions

Although the gluco-conjugate was designed to delay the absorption of thymol, TαG supplementation did not result in higher thymol concentrations in the gut of weaned piglets. Luminal concentrations in the stomach were even lower than with the addition of free thymol. Additionally, thymol-treated animals suffered less from diarrhea, showed an improved jejunal barrier function, and reduced mucosal β-glycosidase activities in the duodenum when compared to control piglets. Conversely, gluco-conjugation reduced the biological effects of thymol in vivo. It can be concluded that the conjugation of thymol into a single glucose molecule was insufficient to obtain the desired antimicrobial effect in the small intestine.

## Figures and Tables

**Table 1:** Ingredient and analyzed nutrient composition of the basal diet (g/kg as fed).

Compounds	Amount	Compounds	Amount
**Ingredient Composition**		**Calculated Nutrient Composition**	
Wheat	297	NEv for pigs (MJ/kg)	10.0
Barley	250	Digestible ^2^ Lysine	11.4
Corn	150	Digestible ^2^ methionine + cysteine	6.8
Soybean meal	76	Digestible ^2^ threonine	7.4
Whey powder	53	Digestible ^2^ tryptophan	2.4
Toasted soybeans	50	Calcium	6.6
Sunflower meal	31	Digestible phosphorus	3.1
Potato protein	30	Sodium	2.9
Soybean oil	23	Potassium	7.1
Limestone (fine)	11	Chlorine	4.0
Sodium bicarbonate	5.6	Magnesium	1.4
Dicalcium phosphate	5.3		
Premix minerals and vitamins ^1^	5		
Salt	2.0	**Analyzed Nutrient Composition**	
L-Lysine	5.4	Dry matter	896
L-Threonine	2.3	Crude ash	47
DL-Methionine	1.9	Crude protein	174
L-Valine	1.1	Crude fat	55
L-Tryptophan	0.7	Crude fibre	36
Phytase (10,000 FYT/g)	0.1		

^1^ Providing per kg of diet: vit A (retinyl acetate), 10,000 IU; vit D3 (cholecalciferol), 2000 IU; vit E (dl-a-tocopherol), 40 mg; vit K3 (menadione), 1.5 mg; vit B1 (thiamin), 1.0 mg; vit B2 (riboflavin), 4.0 mg; vit B6 (pyridoxin hydrochloride), 1.5 mg; vit B12 (cyanocobalamin), 20 μg; niacin, 30 mg; D-pantothenic acid, 15 mg; choline chloride, 150 mg; folic acid, 0.4 mg; biotin, 0.05 mg; Fe (iron(II)sulphate monohydrate), 100 mg; Cu (copper(II)sulphate pentahydrate), 20 mg; Zn (Zinc(II)sulphate monohydrate), 70.0 mg; Mn (manganese(II)oxide), 30 mg; I (potassiumjodate anhydrate), 0.7 mg; Se (sodium selenite), 0.25 mg; ^2^ expressed as apparent ileal digestibility.

**Table 2:** The effect of dietary treatment on the animal performance of piglets from d0 to d13 post-weaning.

	T_CON_^1^	T_THY_	T_TαG_	SEM	*p*-Value
Body Weight (kg)					
d0	6.52	6.52	6.52	0.23	0.999 ^2^
d5	6.24	6.56	6.59	0.10	0.294
d13	8.11	8.41	8.56	0.17	0.550
Average Daily Gain (g/d)					
d0–5	−46	7	12	13.7	0.199
d5–13	234	231	246	14.1	0.894
d0–13	114	135	146	11.8	0.550
Average Daily Feed Intake (g/d)					
d0–5	62	79	87	9.0	0.539
d5–13	262	274	295	17.2	0.741
d0–13	185	199	215	13.4	0.673
Feed:Gain					
d0–5	−0.88	5.06	−4.67	1.62	0.082
d5–13	1.14	1.19	1.20	0.04	0.811
d0–13	1.54	1.48	1.50	0.04	0.823
Fecal Consistency					
Average Score ^3^	1.81 ^a^	1.53 ^b^	1.60 ^ab^	0.20	0.038
Diarrhea incidence (%)	24.3	12.2	20.3	2.1	0.086

^a,b^ Least square means within a row without a common superscript letter differ significantly (*p* ≤ 0.05). ^1^ T_CON_: basal diet; T_THY_: basal diet supplemented with 3.7 mmol thymol/kg DM; T_TαG:_ basal diet supplemented with 3.7 mmol thymol-α-D-glucopyranoside/kg DM; *n* = 6. ^2^ Welch correction used. Calculated means are displayed. ^3^ Expressed on a scale from 1 (normal feces) to 3 (diarrhea).

**Table 3:** The effect of dietary treatment on barrier function and histo-morphology in the distal jejunum of piglets between 13 and 15 days post-weaning.

	T_CON_^1^	T_THY_	T_TαG_	SEM	*p*-Value
Apparent Permeability (*10^−7^ cm/s)^2^					
FD4 ^3^	6.54 ^a^	2.88 ^b^	6.32 ^ab^	0.84	0.028 ^4^
HRP ^3^	3.02	0.67	1.31	0.47	0.143 ^4^
Histo-Morphology ^2^					
Villus Height (µm)	513	584	548	15	0.160
Crypt depth (µm)	205	205	200	3	0.715
Villus height:crypt depth	2.57	2.88	2.77	0.06	0.071

^a,b^ Least square means within a row without a common superscript letter differ significantly (*p* ≤ 0.05). ^1^ T_CON_: basal diet; T_THY_: basal diet supplemented with 3.7 mmol thymol/kg DM; T_TαG:_ basal diet supplemented with 3.7 mmol thymol-α-D-glucopyranoside/kg DM; ^2^ *n* = 8 for permeability, *n* = 12 for histomorphology. ^3^ FD4 = fluorescein isothiocyanate–dextran 4-kDa; HRP = horseradish peroxidase 40-kDa. ^4^ Welch correction used. Calculated means are displayed.

**Table 4:** The effect dietary treatment on bacterial counts (log_10_ CFU/g) in the small intestine of piglets between 13 and 15 days post-weaning.

	T_CON_^1^	T_THY_	T_TαG_	SEM	*p*-Value
SI1 ^2^					
Lactobacilli	7.38	7.36	7.46	0.09	0.898
Total anaerobes	6.60	6.72	6.39	0.14	0.334 ^3^
*E. coli*	3.44 ^ab^	3.35 ^b^	4.29 ^a^	0.16	0.041
Total coliforms	4.21	3.33	3.81	0.22	0.267
Streptococci	7.31	7.45	7.41	0.09	0.820
SI3					
Lactobacilli	8.71	8.38	8.85	0.08	0.070
Total anaerobes	7.53	7.07	7.44	0.09	0.069
*E. coli*	5.51	5.26	6.22	0.17	0.078
Total coliforms	5.48	5.22	6.21	0.17	0.069
Streptococci	8.44	8.45	8.79	0.09	0.174

^a,b^ Least square means within a row without a common superscript letter differ significantly (*p* ≤ 0.05). ^1^ T_CON_: basal diet; T_THY_: basal diet supplemented with 3.7 mmol thymol/kg DM; T_TαG:_ basal diet supplemented with 3.7 mmol thymol-α-D-glucopyranoside/kg DM; *n* = 12. ^2^ SI1 and SI3 correspond to segments of 0–25% and 75–100% of the total small intestinal length, respectively. ^3^ Welch correction used. Calculated means are displayed.

**Table 5:** The effect of dietary treatment on bacterial metabolites (µmol/g) in the small intestine and mid-colon of piglets.

	T_CON_^1^	T_THY_	T_TαG_	SEM	*p*-Value
SI3 ^2^					
Total Volatile Fatty Acids	6.44	7.47	5.65	0.63	0.504
% Acetate	96.6	98.2	98.3	0.6	0.330 ^3^
% Propionate	3.2	1.3	1.5	0.6	0.132 ^3^
% Butyrate	0.3	0.6	0.2	0.1	0.349 ^3^
Lactate	53.45	42.42	61.30	5.04	0.320
Mid-colon					
Total Volatile Fatty Acids	164.9	176.0	162.1	3.55	0.251
% Acetate	53.7	55.4	55.0	0.7	0.505 ^4^
% Propionate	27.0	26.2	27.3	0.6	0.699
% Iso-butyrate	1.0	1.1	0.7	0.1	0.182
% Butyrate	13.2	13.3	12.4	0.4	0.703
% Iso-valerate	1.4	1.5	1.2	0.1	0.254 ^4^
% Valerate	3.8	2.7	3.3	0.2	0.088
Lactate	22.14	23.53	21.08	0.53	0.185

^1^ T_CON_: basal diet; T_THY_: basal diet supplemented with 3.7 mmol thymol/kg DM; T_TαG:_ basal diet supplemented with 3.7 mmol thymol-α-D-glucopyranoside/kg DM; *n* = 12. ^2^ SI3 corresponds to a segment of 75–100% of the total small intestinal length. ^3^ Kruskal-Wallis non-parametric test. Calculated means are displayed. ^4^ Welch correction used. Calculated means are displayed.

**Table 6:** The effect of dietary treatment on the glycosidase activity in the mucosa (mmol/min/g) and digesta (nmol/min/g) of piglets.

	T_CON_^1^	T_THY_	T_TαG_	SEM	*p*-Value
Mucosa (mmol/min/g)					
α-Glucosidase					
Duodenum	0.84	0.87	0.87	0.03	0.863
Proximal Jejunum	1.17	1.06	1.25	0.06	0.399 ^3^
Distal Jejunum	1.06	1.10	1.29	0.06	0.350 ^3^
β-Glucosidase					
Duodenum	12.03 ^a^	7.95 ^b^	11.25 ^ab^	0.62	0.027
Proximal Jejunum	11.93	9.31	10.54	0.67	0.299
Distal Jejunum	4.88	4.66	4.41	0.39	0.882
β-Galactosidase					
Duodenum	18.93 ^a^	13.25 ^b^	17.68 ^ab^	0.85	0.026
Proximal Jejunum	21.23	17.59	18.40	0.95	0.276
Distal Jejunum	14.39	15.70	18.59	1.06	0.269
Digesta (nmol/min/g)					
α-Glucosidase					
Stomach	25.0	24.2	17.7	1.8	0.212
SI1^2^	113.1	67.4	113.2	11.5	0.190
SI2	610.1	473.3	578.4	46.7	0.508 ^4^
SI3	1303.0	1097.5	1248.4	63.2	0.348
Caecum	479.7	357.0	433.0	53.9	0.596
Mid-colon	441.7	450.0	545.6	40.4	0.489
β-Glucosidase					
Stomach	39.1	36.6	27.3	3.2	0.271 ^3^
SI1	325.0	290.3	319.8	38.9	0.926
SI2	1276.1	1033.9	1153.7	124.8	0.643 ^4^
SI3	1852.5	1684.1	1942.5	202.2	0.863
Caecum	674.8	564.8	663.2	98.8	0.650 ^4^
Mid-colon	838.1	585.8	512.1	81.3	0.280 ^3^
β-Galactosidase					
Stomach	22.6	22.2	19.8	2.0	0.829
SI1	264.1	238.0	276.6	31.3	0.862
SI2	1132.4	953.9	963.6	113.5	0.715 ^4^
SI3	1673.0	1396.6	1831.3	186.9	0.619
Caecum	1341.0	1583.2	1715.3	246.9	0.818
Mid-colon	1846.9	1815.6	2103.6	144.4	0.668

^a,b^ Least square means within a row without a common superscript letter differ significantly (*p* ≤ 0.05). ^1^ T_CON_: basal diet; T_THY_: basal diet supplemented with 3.7 mmol thymol/kg DM; T_TαG:_ basal diet supplemented with 3.7 mmol thymol-α-D-glucopyranoside/kg DM; *n* = 12. ^2^ SI1, SI2, and SI3 correspond to segments of 0–25%, 25–75%, and 75–100% of the total small intestinal length, respectively. ^3^ Welch correction used. Calculated means are displayed. ^4^ Kruskal-Wallis non-parametric test. Calculated means are displayed.

## Supplementary Materials

The following are available online at https://www.mdpi.com/2076-2615/10/2/329/s1, Figure S1: Schematic overview of the allocation; Figure S2: Bacterial community compositions present in the digesta of the last 25% of the small intestinal length for each individual pig. The cumulated histograms show the relative abundance of the identified taxa at the (**a**) phylum, (**b**) family, and (**c**) genus levels.
