# Supplementary material for: Effects of Thymol and Thymol α-D-Glucopyranoside on Intestinal Function and Microbiota of Weaned Pigs
Source: Animals (Basel). 2020 Feb 19;10(2):329. doi: 10.3390/ani10020329 (PMC7070699; doi:10.3390/ani10020329)
Supplement: Supplementary file 1 [file animals-10-00329-s001.pdf]

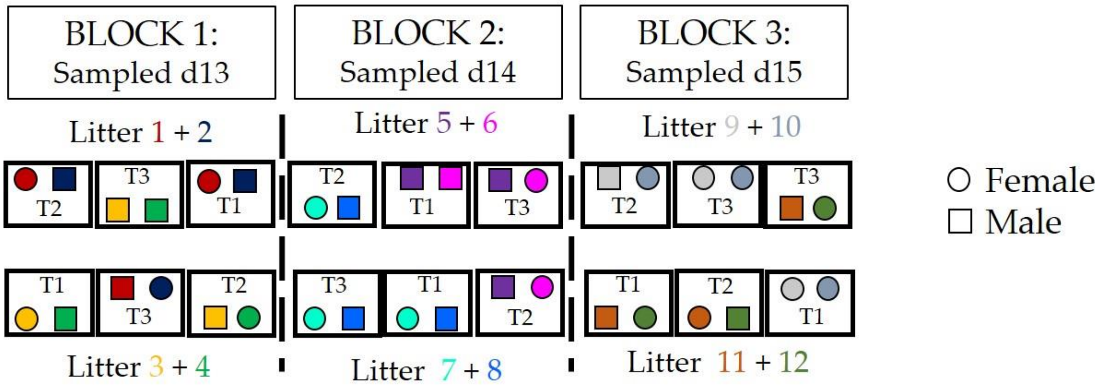

Figure S1: Schematic overview of the allocation

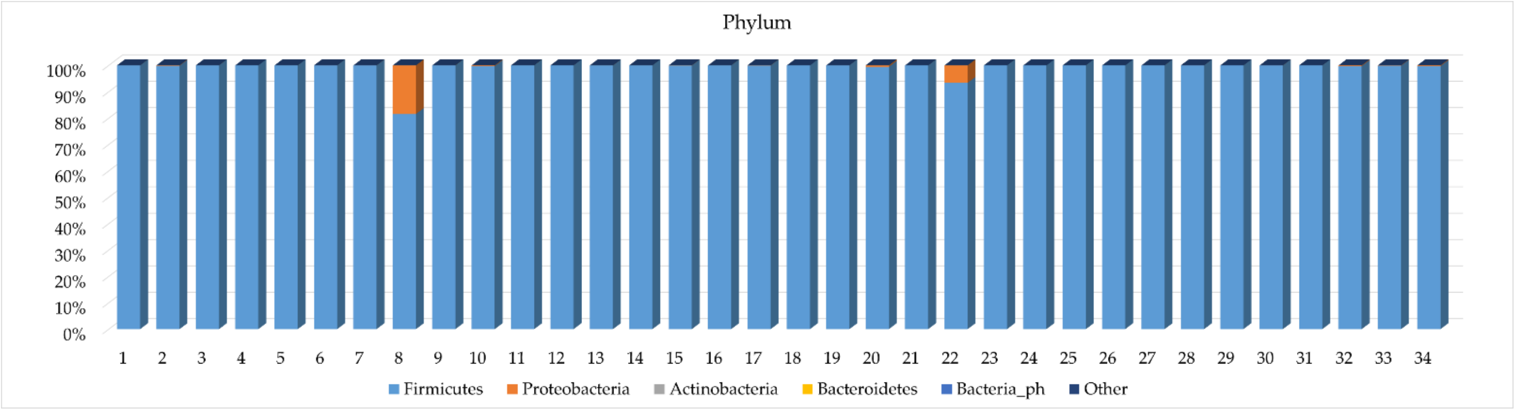

(a)

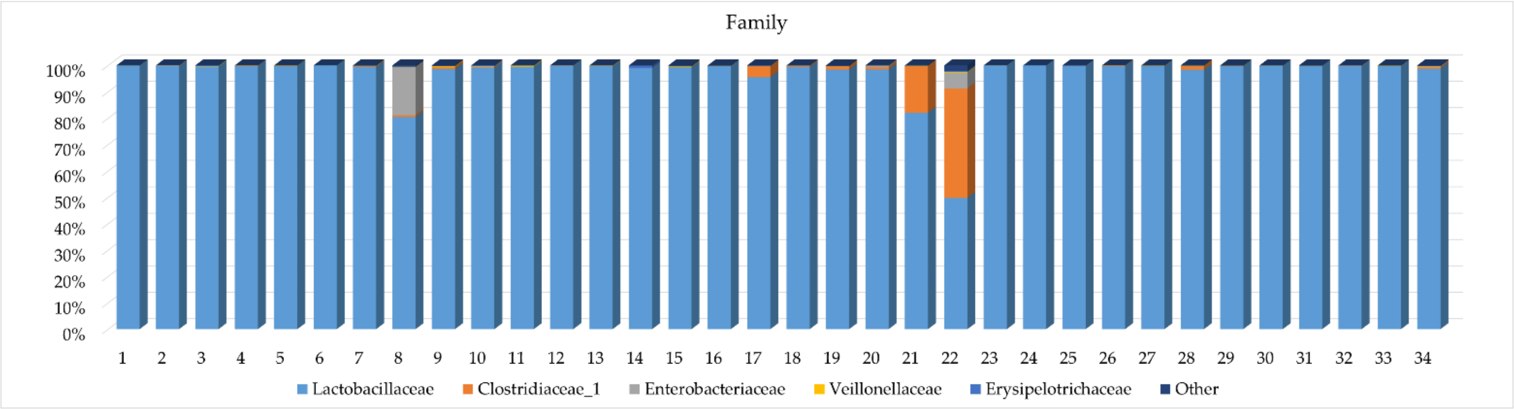

(b)

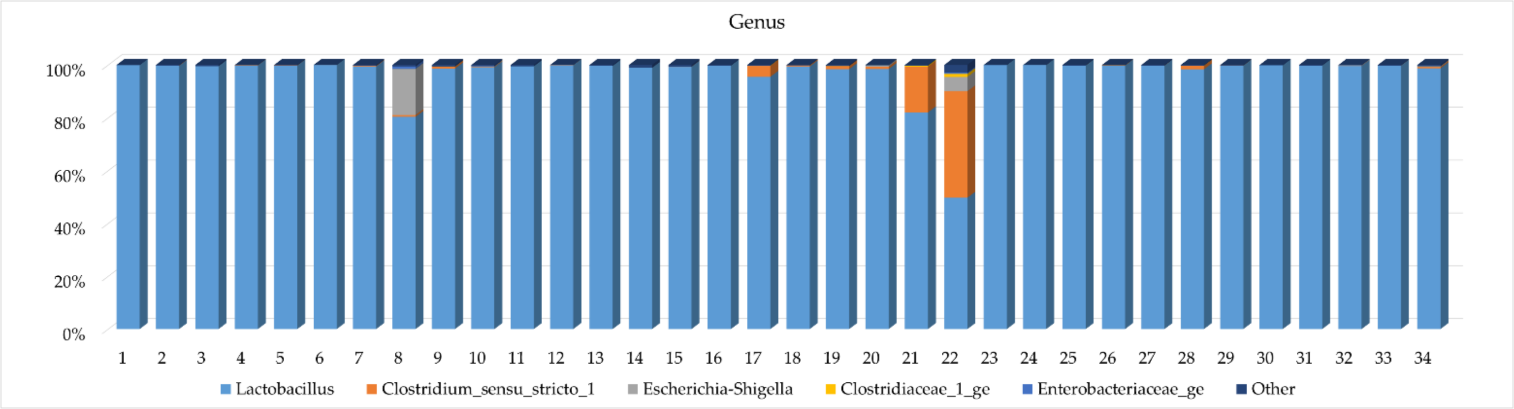

(c)

Figure S2: Bacterial community compositions present in digesta of the last 25% of small intestinal length for each individual pig. The cumulated histograms show the relative abundance of the identified taxa at phylum (a), family (b) and genus (c) level.
